# The effects of blood flow restriction training on PAP and lower limb muscle activation: a meta-analysis

**DOI:** 10.3389/fphys.2023.1243302

**Published:** 2023-11-09

**Authors:** Jian Wang, Haiyang Liu, Lizhu Jiang

**Affiliations:** ^1^ Department of Physical Education, Ningbo University of Technology, Ningbo, China; ^2^ Ningde Vocational and Technical College, Ningde, China

**Keywords:** blood flow restriction training, muscle activation, post-activation potentiation, meta-analysis, lower limb muscle

## Abstract

**Objective:** This study aims to systematically evaluate the effects of blood flow restriction (BFR) training on lower limb muscle activation and post-activation potentiation (PAP) in athletes through a meta-analysis and discuss methods to improve instant muscle strength so as to provide a reference for training in this field.

**Methods:** Randomized controlled trials (RCTs) that examined the impact of BFR training on muscle activation and PAP were gathered through database searches, such as CNKI, Wanfang, Web of Science, PubMed, and others. The Cochrane risk of bias tool was used to include and exclude literature. Quality evaluation and statistical analysis were conducted using ReviewManager 5.3 software, STATA 16.0, and other software programs. The sensitivity analysis and funnel plots were employed to assess result stability and publication bias.

**Results:** In total, 18 literature studies were included with a total of 267 subjects. The meta-analysis showed that BFR could significantly improve the RMS value of lower limb muscles [*SMD* = 0.98, 95% *CI* (0.71, 1.24), and *p* < 0.00001]. BFR had a significant effect on the immediate explosive power of the lower limbs [*SMD* = 0.28, 95% *CI* (0.02, 0.53), and *p* = 0.03], but the heterogeneity was obvious (*I* ^2^ = 51%). The subgroup analysis showed that different training methods may be influencing factors that lead to the heterogeneity between studies. The measurement indexes were the counter movement jump (CMJ) [*SMD* = 0.45, 95% *CI* (0.20, 0.69), and *p* = 0.0004], training mode to overcome body weight [*SMD* = 0.57, 95% *CI* (0.33, 0.82), and *p* < 0.00001], and compressive strength of 40%–60% arterial occlusion pressure (AOP) [*SMD* = 0.57, 95% *CI* (0.31, 0.83), and *p* < 0.0001], which reached the maximum effect and was statistically significant.

**Conclusion:** BFR training can induce lower extremity muscle activation and PAP. Combining self-weight training with BFR exercises set at 40%–60% AOP appears to be particularly effective in inducing PAP, especially for enhancing CMJ. Furthermore, combining body-weight training with BFR is considered an effective warm-up method to improve CMJ.

**Systematic Review Registration**: http://inplasy.com, identifier INPLASY2023100087

## 1 Introduction

With increasing competitiveness in sports, traditional training methods often fall short of meeting athletes’ demands for enhancing their competitive abilities. Developing explosive muscle power is a common objective for athletes participating in disciplines such as jumping, throwing, or sprinting. During their training, athletes often incorporate specialized exercises tailored to the unique characteristics of their sport, which may include augmentation or resistance exercises. Researchers have discovered that blood flow restriction (BFR) training can serve as a supplementary approach to high-intensity resistance training, effectively improving muscle contraction function and promoting muscle strength growth (Xu and Wang, 2013). It achieves this by applying pressure to the outer regions of the limbs, which causes occlusion of venous blood flow at the distal ends of the limbs. Subsequently, training is conducted at lower exercise intensities, thereby facilitating optimal muscle activation during the warm-up (Che et al., 2021). Studies have indicated that the combination of BFR training with low-intensity resistance training can improve the recruitment capacity of the lower limb muscles (Wang, 2021).

As a method for rapidly enhancing strength, post-activation potentiation (PAP) is achieved through controlled training, such as squats and deadlifts, which induce intense neuromuscular excitement and, consequently, rapidly improving muscle explosiveness within a short timeframe (Batista Mauro et al., 2011). High-intensity exercise is achieved through the rapid recruitment of muscle fibers by the nervous system. Consequently, enhancing muscle nerve activation before a competition holds significant importance for improving sports performance (Lin et al., 2017; Xu et al., 2020). Nevertheless, there is controversy surrounding the induction mode and timing of PAP. Most coaches tend to use maximum resistance exercise to induce PAP (Hanson et al., 2007; Mccann and Flanagan, 2010). However, this high-intensity and high-load training approach can lead to sports injuries and muscle fatigue. By contrast, BFR training is a low-energy consumption, high-efficiency exercise mode. By restricting blood flow to the limbs, metabolites accumulate, prompting the body to falsely simulate stress in muscle fibers by intensive exercising, thereby optimizing training (Hu, 2020).

Warm-up exercises have been extensively researched, and scientific warm-up routines can effectively enhance athletic performance. Studies have shown that the PAP effect can optimize warm-up programs for endurance sports, resulting in improved athletic achievements (Wu et al., 2020). However, the potential of utilizing BFR to enhance warm-up effects remains largely unexplored. In light of this, this study conducts a meta-analysis that summarizes the impact of BFR training combined with different exercise modalities on lower limb muscle activation and PAP, finding the optimal pressure intensity to induce an enhancement effect of lower limb activation and determining the appropriate exercise mode that can be combined with BFR training during warm-up. Our focus lies on the lower limb muscles below the iliac crest (Zhao et al., 2006). The selected experiments are all randomized controlled trials that aim to provide a reference for enhancing athletes’ muscle recruitment capacity and explosiveness.

## 2 Materials and methods

### 2.1 Search strategy

A total of 159 documents were retrieved from the CNKI, Wanfang, VIP, PubMed, and Web of Science databases up to 4 February 2023. The English search terms used were as follows: (“blood flow restriction training” or “BFR” or “KAATSU training” or “pressure training”) and (“Potentiation after activation” or “PAP” or “muscle recruitment” or “explosive power” or “lower extremity”) and (“RCT”).

### 2.2 Inclusion and exclusion criteria

#### 2.2.1 Inclusion criteria

Type of study: All included literature were publicly published and involved randomized controlled trials (RCTs) that studied the effects of BFR training on muscle activation and post-activation enhancement effects. Study subjects: The study subjects included healthy adults, both with and without prior training experience. Interventions: The experimental group received BFR training, while the control group received either other training modalities or no training at all. Outcome measures: The primary outcome measure was related to the quantitative lower limb (below the point of the iliac crest) (Zhao et al., 2006), which included measures of muscle activation and PAP such as MVC moment, EMG value, and longitudinal jump height. Additional criteria: The studies should provide details about the experimental design and intensity of BFR training, among other relevant information. Source inclusion: To minimize the risk of bias in the included literature, this study considers only articles indexed in the SCI (Science Citation Index).

#### 2.2.2 Exclusion criteria

Unclear research type: Studies lacking clear documentation of their research type were excluded. Non–BFR training: Studies that involved interventions other than BFR training were excluded. Duplicate publications: Repeatedly published articles for which full text could not be obtained and review articles were excluded. Lack of quantitative outcome data: Studies without quantitative outcome indicators or valid data were excluded. Animal experiments: Research that involved animal experiments were excluded.

### 2.3 Data Extraction

**FIGURE 1:**
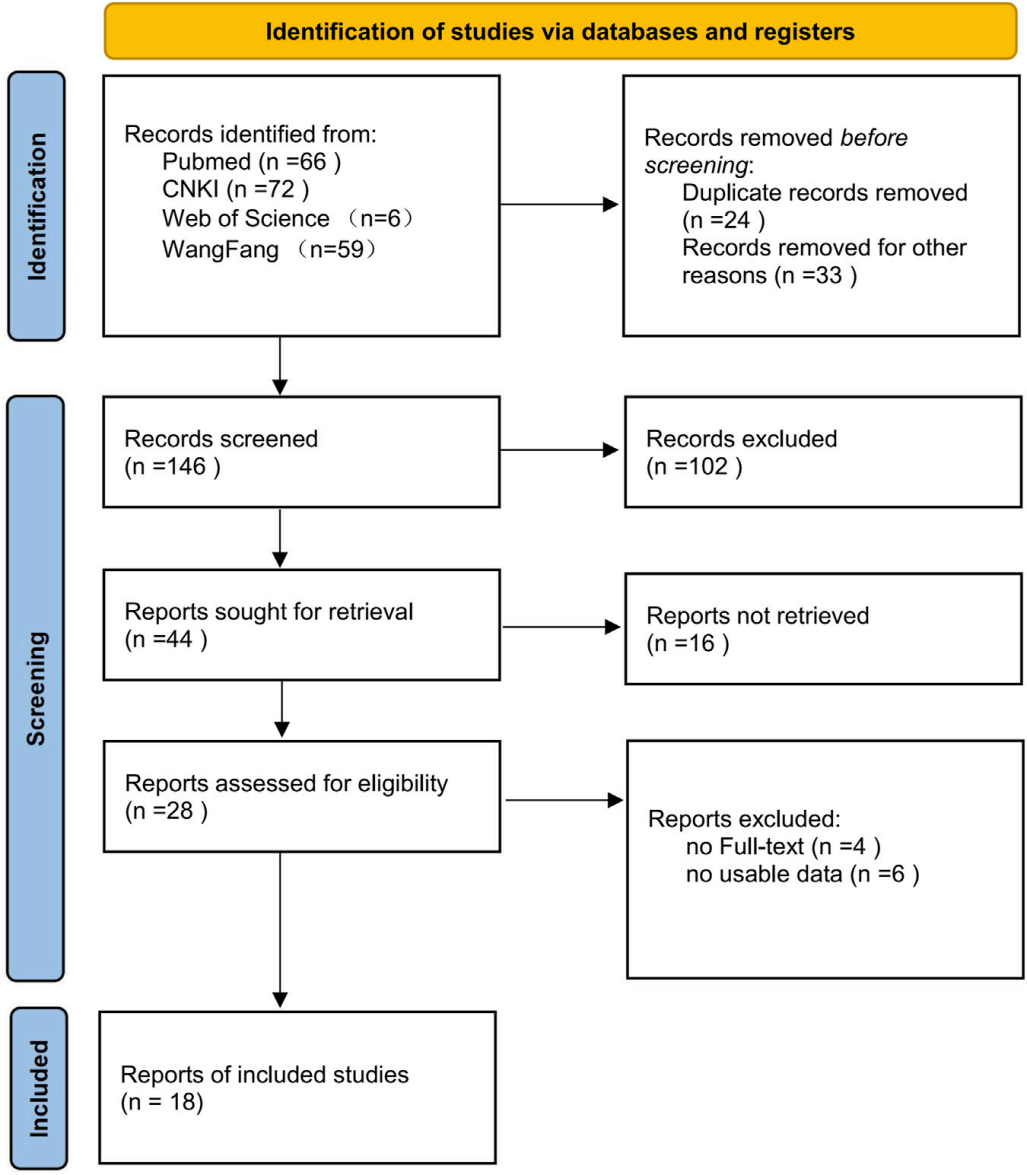
Flow diagram of literature selection.

The collected literature were imported into EndNote software and underwent independent screening by two researchers. The steps of literature screening and inclusion are illustrated in Figure 1. Ultimately, 18 articles were included in this review.

Data extraction: Two researchers independently extracted information according to a custom-made form, which primarily encompassed the following categories:

1. General information: first author and year of publication.
2. Sample information: details about the research subjects such as age and sample sizes for both the experimental and control groups.
3. Characteristics of exercise intervention: information on intervention measures for both the experimental and control groups, and specifics of the intervention programs for the experimental group (such as training methods, training volume, and training intensity).
4. Outcome indicators: quantitative measures of lower limb muscle activation and test indicators related to PAP.

### 2.4 Statistical analysis

Statistical analysis was performed using ReviewManager 5.3 software. The outcome indicators included in the literature in this study were continuous variables, and standardized mean differences (*SMDs*) and 95% confidence intervals were chosen for effect sizes because of the different testing methods used for each indicator. The Cochrane risk bias assessment tool was referenced for literature quality assessment. The homogeneity test (Q test, test level a = 0.1) was used to test for heterogeneity, and *I* ^2^ values from 0% to 100%, *I* ^2^ > 50%, and *P* < *a* indicated the existence of heterogeneity, and the random effects model was selected. The random effects model was chosen for meta-analysis, while the fixed effects model was chosen for the opposite. The subgroup analysis was used for the treatment of heterogeneity and STATA 16.0 was used for sensitivity analysis to test the stability of the results. The Egger test and funnel map were used to check for the presence of publication bias.

## 3 Results

### 3.1 Study characteristics

A total of 18 publications were included in this study, all of which were RCTs, which included 267 subjects with mixed genders and an age range of 18–33 years, with the basic characteristics shown in Table 1.

**TABLE 1:** Characteristics of studies included in the systematic review and meta-analysis.

Study	Country	Age (years)	N (EG/CG)	Intervention (EG/CG)	Plan (BFR intensity)	Outcome extracted
Wang (2021)	China	19.65 ± 0.94	19/19	BFR/No BFR	Four groups of 30-15-15-15 times 30% 1RM knee extension resistance (40%)	RMS VM↑
						MVC↓
Li et al. (2021)	China	20.3 ± 1.9	10/10	BFR/No BFR	Four groups of 30-15-15-15 times 30% 1RM knee extension resistance (40%)	RMS VM↑
Hu (2020)	China	18.3 ± 3.28	12/12	BFR/HRT, LRT	Two groups of 30 + 20 30% 1RM squats (bundled pressure 40 mmHg and inflation pressure 200 mmHg)	RMS VM↑
						CMJ↑
						MVC↑
Li (2020)	China	21.4 ± 3.2	10/10	BFR/No BFR	Four sets of 30-15-15-15 squats (40%)	RMS RF NS
Hongwen and Xiang (2022)	China	23.6 ± 1.51	27/27	BFR/No BFR	Two sets of 10 straight leg jumps + three sets of five consecutive obstacle jumps + five drop jumps (inflation pressure 160 mmHg)	CMJ↑
						RCMJ NS
Yan and Guo (2018)	China	20.3 ± 2.3	20/20	BFR/No BFR	Five sets of 50% HRR and 2-min interval runs (inflation pressure 150 mmHg)	CMJ↑
						MVC↑
Zhang (2021)	China	15.83 ± 1.0	12/12	BFR/No BFR	20 min cycling (inflation pressure 250 mmHg)	P(W) 10 s↑
Che et al. (2021)	China	17.57 ± 2.83	10/10	BFR/No BFR	Four groups of 30-15-15-15 times 30% 1RM half squats (bundled pressure 40 mmHg and inflation pressure 180 mmHg)	RMS GM↑
Sun et al. (2019)	China	33.5 ± 4.9	10/10	BFR/No BFR	Four groups of 30-15-15-15 times 20% 1RM hard pull (20 mmHg)	RMS BF↑
Pan et al. (2019)	China	21.14 ± 1.17	18/18	BFR/No BFR	Four groups of 30-15-15-15 1RM knee extension resistance exercises (40%, 60%, and 80%)	sEMG (uV)↑
Amani et al. (2019)	Iran	23 ± 2	6/6	BFR/No BFR	Three-player football training with BFR (80–100 mmHg)	RMS VM↑
						MVC↑
Yun-Tsung et al. (2019)	China	22.7 ± 4.6	12/12	BFR/No BFR	Five sets of 50% HRR and 2 min runs (149.8 ± 5.0 mmHg)	MVC↑
						RMS BF↑
Cleary Christopher and Cook Summer (2020)	United States	20.3 ± 0.9	15/15	BFR/No BFR	One set of thirty 30% 1RM back squats (60%)	VJ NS
						RMS VM NS
Doma et al. (2020)	Australia	22.9 ± 5.0	18/18	BFR/No BFR	Three sets of eight single-leg lunges, leg swings, high knees, and hip kicks each (130% systolic pressure)	VJ NS
						MJ↑
Pedro et al. (2016)	Portugal	24.8 ± 5.4	14/14	BFR/No BFR	Six groups each with 10 maximum knee extension eccentric movements (40%)	RMS RF↑
						MVC↓
Gepfert et al. (2020)	Poland	28.4 ± 5.8	10/10	BFR/No BFR	Three sets of three 70% 1RM barbell squats (100%)	P(W)↑
						V↑
Lauver et al. (2017)	United States	22.8 ± 2.3	24/24	BFR/No BFR	Four groups of 30-15-15-15 20% 1RM knee resistance exercises (130% systolic blood pressure)	MVIC↓
						RMS VM↑
Miller et al. (2018)	United States	21.8 ± 2.6	20/20	BFR/No BFR	Three sets of 20-s WBV	CMJ↑
					Three sets of MVC (charging pressure 160 mmHg)	

NS, no statistical significance; RMS, electromyographic standard value; MVC, maximum autonomous isometric contraction; VM, medial thigh muscle; RF, rectus femoris muscle; GM, gluteus maximus muscle; BF, biceps femoris muscle; R, rate. ↑ represents a significant increase; ↓ represents a significant decrease; WBV, whole body vibration; HRR, heart recovery rate; CMJ, counter movement jump; MVIC, maximal voluntary isometric contraction; V, velocity of Bench Press; P(W), maximum output power; RCMJ, rate of counter movement jump.

### 3.2 Study quality assessment

**FIGURE 2:**
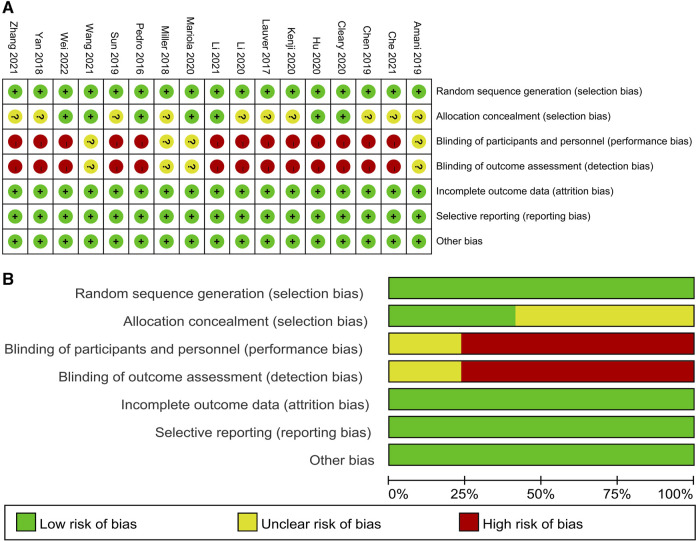
Methodological quality graph and summary of the included studies: **(A)** risk of bias summary and **(B)** risk of bias graph.

The quality of the collected literature was evaluated with reference to the Cochrane risk of bias assessment tool (Higgins and Altman, 2007). Review Manager 5.3 software was used to assess seven aspects, namely, the random allocation method, allocation scheme concealment, participant blinding, outcome blinding, completeness of outcome data, selective reporting of study results, and other sources of bias (Figures 2A, B). Twelve articles did not clearly describe whether the implementer of the assignment had strictly performed the random assignment, and 18 articles had a high risk of bias due to informed consent being signed before the experiment. Thus, there was a high risk of bias in the blinding.

### 3.3 Lower limb muscle activation

**FIGURE 3:**
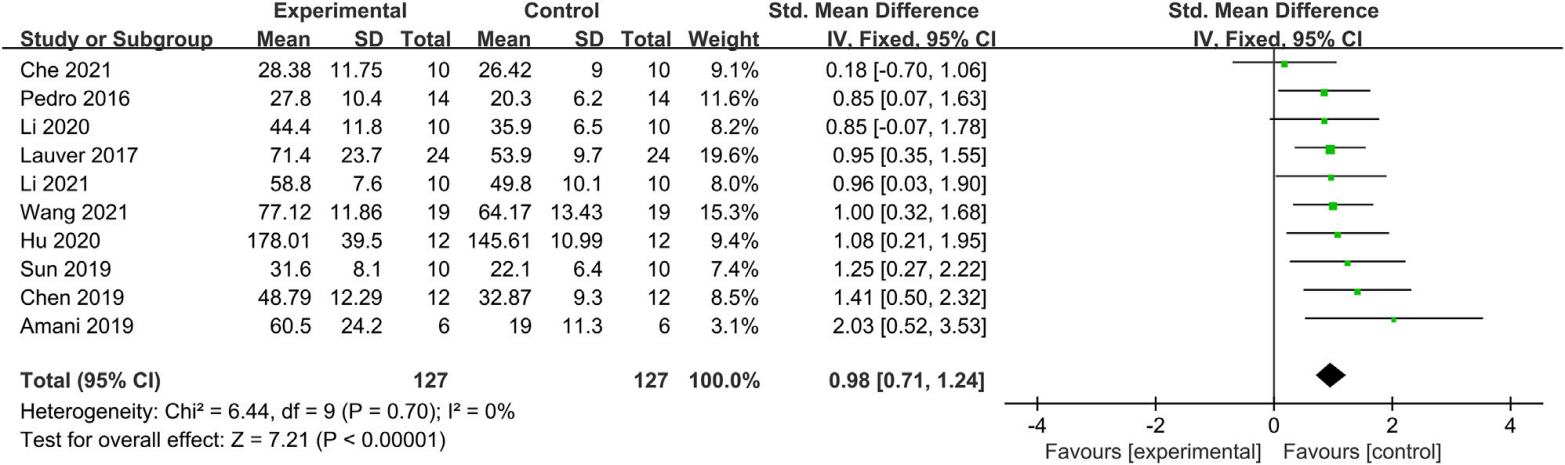
Effect of BFR training on neuromuscular activation.

Out of the 18 included articles, 10 compared 127 subject differences in RMS standard values before and after BFR training (Figure 3). After testing for heterogeneity, *I* ^2^ = 0.0% < 50% and *p* = 0.70 > 0.1 for the Q test, suggesting no heterogeneity among the included literature. The fixed effects were selected for the meta-analysis. The *SMD* value for the pooled 10 studies was 0.98, with a 95% confidence interval from 0.71 to 1.224 and statistically significant, with *Z* = 7.21 and *p* ≤ 0.05, indicating that BFR exercises can significantly improve RMS values in lower limb muscles.

### 3.4 PAP

**FIGURE 4:**
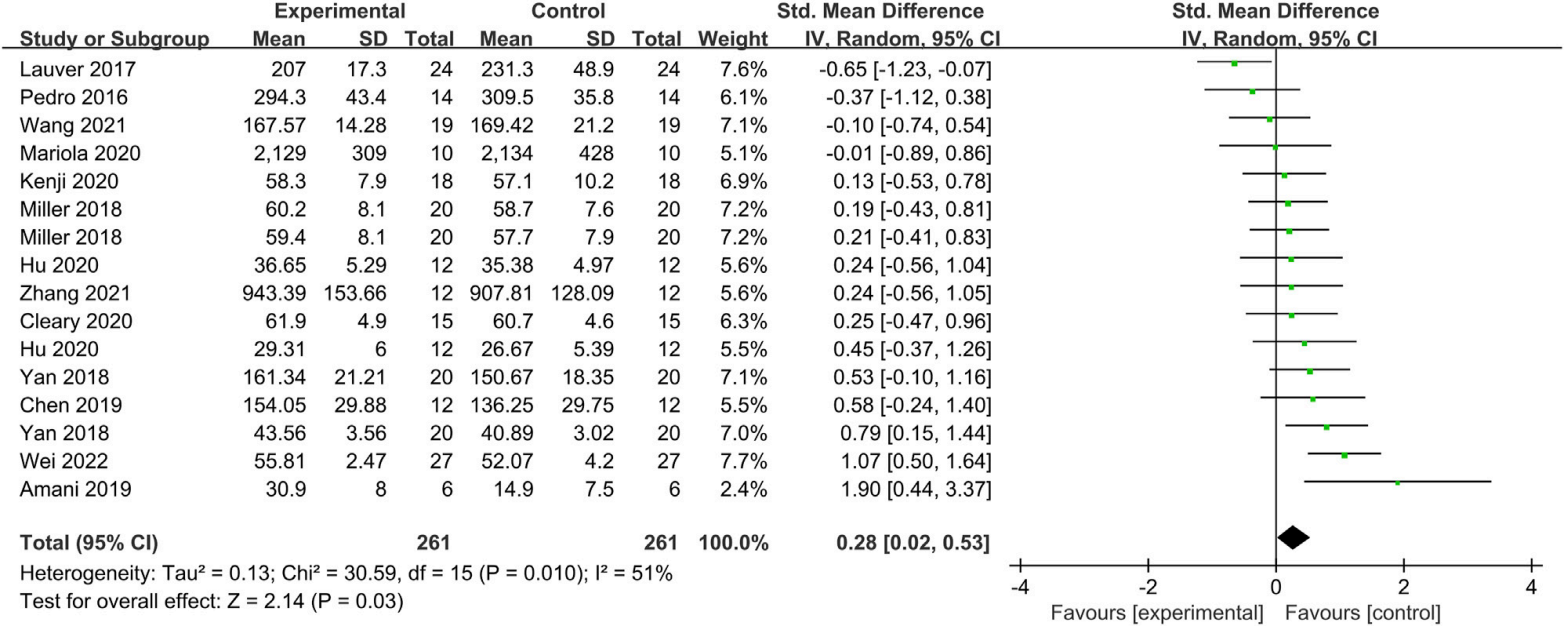
Forest plot of the impact of BFR training on PAP.

For BFR training–induced PAP, 13 articles were included (16 studies with a total of 261 subjects) and were tested for heterogeneity, and *I* ^2^ = 51% > 50% and Q-test of *p* = 0.01 < 0.1, indicating a strong heterogeneity among the included literature, and random effects models were selected. A meta-analysis was performed (Figure 4) and the results showed that the combined effect size was *SMD* = 0.28 and statistically significant (*Z* = 2.13 and *p* = 0.03), which indicates that BFR training could significantly induce the production of PAP when compared with the control group.

### 3.5 Subgroup analysis

Based on the data of this study, the authors suspect that the source of heterogeneity may be the inconsistency of PAP effect test indicators. Therefore, according to the test indicators of the PAP effect (MVC: torque, CMJ: longitudinal jump, and P(W): output power), 13 articles (16 studies) were divided into three groups for the subgroup analysis (Table 2). According to the exercise modality, the included studies were divided into three subgroups: ≤30% 1RM resistance exercises, ≥70% 1RM resistance exercises, and self-weight exercises. For compressive strength, the subgroups were categorized based on the arterial occlusion and thigh circumference, according to Loenneke Jeremy et al. (2015), and three subgroups were identified: 40% AOP and below, 40%–60% AOP, and 60% AOP and above.

**TABLE 2:** Subgroup analysis of lower limb PAP induced by BFR training.

Research feature	Subgroup standard	Study (sample)	*SMD*	95% *CI*	*P*	*I* ^2^ (%)	*P* (heterogeneity)
Outcome extracted	MVC	7 (107)	0.07	−0.21,0.34	0.63	66	0.07
	CMJ	7 (132)	0.45	0.20,0.69	0.0004	28	0.21
	P(W)	2 (22)	0.13	0.47,0.72	0.68	0	0.67
Exercise mode	≤30% 1RM	5 (82)	−0.06	−0.37,0.25	0.69	40	0.15
	≥70% 1RM	3 (44)	−0.02	−0.44,0.39	0.91	0	0.51
	Self-weight	8 (135)	0.57	0.33,0.82	<0.0001	33	0.17
Compressive strength	≤40% AOP	4 (67)	−0.12	−0.47,0.23	0.50	73	0.01
	40%–60% AOP	6 (119)	0.57	0.31,0.83	<0.001	19	0.29
	≥60% AOP	6 (75)	0.13	−0.21.0.45	0.44	0	0.75

The subgroup analysis of the outcome indicators showed that the heterogeneity of the three groups was 66%, 28%, and 0%. When compared with the overall combined effect (*I* ^2^ = 51%),the within-group heterogeneity of the MVC moments had increased to 66%, which suggests strong heterogeneity among the studies with respect to the outcome indicator of the MVC moment. The heterogeneity within the literature on the outcome indicator of the MVC moment was strong. The CMJ longitudinal jump group had the highest effect size and was statistically significant (*SMD* = 0.45 and *p* = 0.0004 < 0.01). The available results suggest that BFR training can significantly improve CMJ performance. The available results also suggest that BFR training can significantly increase the vertical jump height of CMJ.

The subgroup analysis of exercise patterns showed that the heterogeneity of the three groups was 40%, 0%, and 33%, which is lower than the overall combined effect (*I* ^2^ = 51%). It is speculated that different exercise methods may be the influencing factors that lead to the heterogeneity between studies. When compared to resistance exercises at 30% 1RM and below, as well as resistance exercises at 70% and above, self-weight exercise had the largest effect, with an *SMD* of 0.57, and it was statistically significant (*p* < 0.01). This indicates that BFR training combined with self-weight exercise can significantly induce the generation of PAP.

The subgroup analysis of pressure intensity revealed varying degrees of heterogeneity among the three groups: 73%, 19%, and 0%, respectively, in comparison to the overall combined effect (*I* ^2^ = 51%). Notably, within the subgroup of compressive strength of 40% AOP and below, the intra-group heterogeneity increased to 73% (*I* ^2^ = 73%), which indicates significant heterogeneity among the studies in this category. Among these, the effect size of the 40%–60% AOP group was the highest and statistically significant (*SMD* = 0.57 and *p* < 0.01), which suggests that the pressurization intensity of BFR training at 40%–60% AOP significantly induces PAP production.

### 3.6 Sensitivity analysis

Sensitivity analysis was conducted on the included literature, both by including and excluding individual groups of studies, to assess heterogeneity.

In Table 3, the combined effect of BFR training on lower limb muscle activation, as included in all studies, resulted in an *SMD* of 0.98 (*p* < 0.01 and *I* ^2^ = 0%). The *SMD* range from individual studies was from 0.94 to 1.06, with I^2^ = 0%, and all *p*-values were <0.01. There was no single literature that posed a threat to the meta-analysis results, which indicates the study’s stability.

**TABLE 3:** Combined effects of lower limb muscle activation after excluding individual studies.

Study	*SMD*	95% *CI*	*P* (merge effect)	*I* ^2^ (%)
Che et al. (2021)	1.06	0.78, 1.33	<0.00001	0
Pedro et al. (2016)	0.99	0.71, 1.27	<0.00001	0
Li (2020)	0.99	0.71, 1.26	<0.00001	0
Lauver et al. (2017)	0.98	0.69, 1.28	<0.00001	0
Zhao et al. (2006)	0.98	0.70, 1.25	<0.00001	0
Wang (2021)	0.97	0.68, 1.26	<0.00001	0
Hu (2020)	0.97	0.69, 1.24	<0.00001	0
Sun et al. (2019)	0.95	0.68, 1.23	<0.00001	0
Yun-Tsung et al. (2019)	0.94	0.66, 1.21	<0.00001	0
Amani et al. (2019)	0.94	0.67, 1.21	<0.00001	0
Overall	0.98	0.71, 1.24	<0.00001	0

Table 4 presents the combined effect of BFR training on the PAP effect across all included studies, with an *SMD* of 0.28 (*p* = 0.03 and *I* ^2^ = 51%). After excluding the studies by Hongwen and Xiang (2022) and Lauver et al. (2017), the combined effects were 0.09 and 0.002, respectively, with *I* ^2^ values of 37% and 31%. This resulted in a significant reduction in heterogeneity. The combined effect, after excluding other single studies, had an *SMD* of 0.02–0.07, with *p*-values < 0.1.

**TABLE 4:** PAP merger effect after excluding individual studies.

Study	*SMD*	95% *CI*	*P* (merge effect)	*I* ^2^ (%)
Hongwen and Xiang (2022)	0.20	−0.03, 0.44	0.09	37
Yan and Guo (2018)	0.24	−0.02, 0.50	0.07	50
Yan and Guo (2018)	0.26	−0.01, 0.53	0.06	53
Hu (2020)	0.28	0.01, 0.55	0.04	54
Hu (2020)	0.27	−0.00, 0.54	0.05	54
Wang (2021)	0.31	0.04, 0.58	0.02	52
Zhang (2021)	0.28	0.01, 0.55	0.04	54
Pedro et al. (2016)	0.32	0.06, 0.58	0.02	49
Miller et al. (2018)	0.29	0.01, 0.56	0.04	54
Miller et al. (2018)	0.29	0.01, 0.56	0.04	54
Gepfert et al. (2020)	0.29	0.03, 0.56	0.03	54
Doma et al. (2020)	0.29	0.02, 0.57	0.04	54
Lauver et al. (2017)	0.35	0.13, 0.58	0.002	31
Cleary Christopher and Cook Summer (2020)	0.28	0.01, 0.56	0.04	54
Yun-Tsung et al. (2019)	0.26	−0.01, 0.53	0.06	53
Amani et al. (2019)	0.24	−0.00, 0.48	0.05	46
Overall	0.28	0.02, 0.53	0.03	51

The same literature name refers to different research results included in the same literature.

### 3.7 Publication bias

**FIGURE 5:**
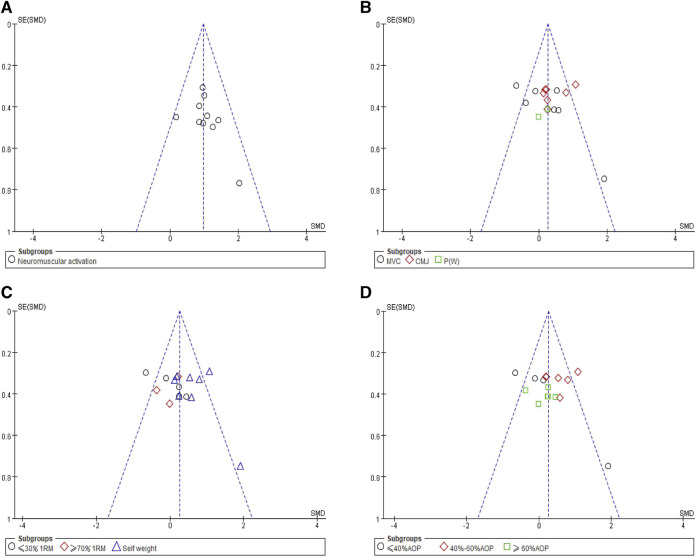
Funnel plots of meta-analysis: **(A)** neuromuscular activation, **(B)** outcome extracted, **(C)** resistance strength, and **(D)** compressive strength.

We conducted bias tests separately by drawing funnel plots within the subgroups to assess the publication bias of the included studies. The funnel plots exhibited symmetry, which indicates no publication bias, as illustrated in Figures 5A–D.

Furthermore, we continued to examine potential bias using the Egger test, and all *p*-values were found to be >0.05, as presented in Table 5. Therefore, it can be concluded that there is no publication bias present in the literature analyzed in this study.

**TABLE 5:** Egger test results.

Research features	Subgroup standard	Coefficient	*t*	*Pr* > |z|
Neuromuscular activation	RMS	1.780	1.30	0.175
Outcome extracted	MVC	5.433	2.53	0.133
	CMJ	−5.578	−1.28	1.000
	P(W)	−7.119	N	1.000
Resistance strength	≤30% 1RM	8.400	4.29	0.086
	≥70% 1RM	−2.553	−0.60	1.000
	Self-weight	1.976	0.96	0.711
Compressive strength	≤40% AOP	5.852	3.43	0.089
	40%∼60% AOP	−5.391	−1.12	1.000
	≥60% AOP	−2.184	−0.76	0.806

N, no value.

## 4 Discussion

### 4.1 BFR training–induced muscle activation in the lower limbs

The induction method employed in this study is BFR training. By combining low-intensity resistance exercises (Lauver et al., 2017; Cleary Christopher and Cook Summer, 2020; Hu, 2020; Li, 2020; Che et al., 2021; Wang, 2021), high-intensity resistance exercises (Pedro et al., 2016; Pan et al., 2019), and aerobic activities such as running (Amani et al., 2019; Yun-Tsung et al., 2019), we assessed their impact on electromyography (EMG) readings of lower limb muscles. As for the criteria for evaluating lower limb muscles, specific sites below the iliac crest point (Wu et al., 2020) were selected, which primarily included the vastus medialis (VM), rectus femoris (RF), gluteus maximus (GM), biceps femoris (BF), and other muscle groups.

Muscle mass and strength development are governed by neural modulation and stimuli (Marcotte et al., 2015). The results of the meta-analysis indicate that the effect sizes from the overall effect tests in all 10 studies were consistently positive (*p* < 0.01). Therefore, BFR training significantly enhances the root mean square (RMS) values of lower limb muscles. The heterogeneity test results indicate complete homogeneity among the study outcomes (*I* ^2^ = 0%), and the sensitivity analysis showed no significant changes in heterogeneity or combined effects when any single study was removed. This suggests that there are no differences related to participant characteristics, exercise methods, pressure intensity, etc., with all included studies pointing to the same outcome.

It is well known that high-intensity exercise is achieved through the recruitment of both fast and slow muscle fibers by the nervous system. Studies have demonstrated a positive correlation between the degree of muscle activation and number of nerve fibers (Westing et al., 1991). When compared to centrifugal exercise alone, combining centrifugal exercise with BFR leads to greater neuromuscular activation (Schoenfeld Brad, 2012). This increased neuromuscular activation observed during BFR exercises is attributed to reduced muscle oxygen availability due to compression, which inhibits *α* motor neurons. To meet the altered energy demands, more type II (fast-twitch) muscle fibers are recruited for participation in the exercise (Yasuda et al., 2015). In the literature within this study, the control group’s intervention measures involved non-pressurized resistance exercises. Therefore, in comparison to traditional resistance exercises, BFR exercises may facilitate the recruitment of more fast-twitch muscle fibers. This recruitment potentially has a positive impact on improving explosive strength and muscle strength in athletes.

### 4.2 Possible mechanism of inducing PAP by BFR training

As a physiological phenomenon characterized by an acute increase in explosive force, methods aimed at inducing PAP are primarily based on heavy-load activities such as squats, bench presses, and high-intensity exercises (Zhuang, 2021). In this study, we conducted a meta-analysis of various explosive force measurement indices, such as MVC moment, CMJ vertical jump height, and P maximum output power, before and after BFR training. The results revealed an overall effect size of 0.28 (*p* < 0.05) across 16 studies, which indicates that BFR training can induce PAP.

Traditional PAP induction methods typically require heavy training equipment for short-term maximum or sub-maximum resistance training, which can pose a risk of sports injuries and subsequent muscle fatigue due to their high-intensity and heavy-load nature. By contrast, BFR training is characterized by low intensity and rapid recovery. Furthermore, creatine kinase (an index representing the degree of muscle damage) did not significantly increase during BFR training, suggesting that muscle fiber damage is lower than it is in high-intensity training (Xu et al., 2021).

In recent years, new warm-up programs based on the PAP effect have gained attention in the academic community. These programs combine resistance training with isometric training to enhance sports performance and have been adopted as pre-match warm-up routines to induce PAP (Yang and Huan, 2022). In summary, BFR training offers a relatively simple and convenient method for effectively inducing the PAP phenomenon when compared to traditional high-intensity resistance exercises.

The production of PAP is primarily attributed to factors such as muscle acidification, strengthening of the H-reflex, and changes in muscle fiber recruitment angles under load stimulation, which result in an increased number of recruited motor units due to motor nerve excitation (Chong et al., 2020). This phenomenon aligns with the neuromuscular activation observed in BFR exercises as mentioned earlier. Cleary Christopher and Cook Summer (2020) also found that EMG amplitudes in activated hamstrings were the highest under BFR conditions during blood restriction training, suggesting a potential link between lower limb muscle activation and BFR training–induced PAP.

#### 4.2.1 Outcome indicators

Following the heterogeneity test (*I* ^2^ = 51%), we conducted a subgroup analysis to explore the reasons for variation among studies, focusing on the outcome indicators related to the PAP effect (Table 2). The results showed that 1) BFR training resulted in a significant increase in height of the CMJ vertical jump; 2) the MVC moment did not exhibit a significant effect on the combined results, which might be attributed to the high heterogeneity in the literature (*I* ^2^ = 66% and *p* = 0.07), with studies using the MVC moment as the outcome indicator, which is the primary source of heterogeneity; and 3) there are only two references on P (output power), and the results of the subgroup analysis also did not reach a significant level.

Three out of the seven studies, which used the MVC moment as the outcome indicator, reported negative effect sizes. The author found that these three studies when compared to the rest of the literature were all conducted on people with no athletic experience. Some researchers suggested that high-level athletes (track and field and rugby) may cause greater PAP than non-athletes (Hamada et al., 2000). Wilson et al. (2013) also demonstrated that athletes exhibited a larger effect size of PAP than non-athletes. The analysis suggests that athletes might display an enhanced response to PAP protocols due to their training adaptations. Therefore, the MVC moment test index in non-athletes may be insensitive to BFR exercises.

As an effective indicator of the PAP effect, CMJ vertical jump height is usually quantified by the following formula (Cleary Christopher and Cook Summer, 2020):

$$\text{PAP}\left(\%\right)=\left[\frac{\text{post}\;\text{CMJ}\;\text{height}\left(\text{cm}\right)}{\text{pre}\;\text{CMJ}\;\text{height}\left(\text{cm}\right)}\times 100\right].$$ (1)

A value >100 indicates the generation of PAP. In the literature comprising seven studies on CMJ longitudinal jump, all reported positive effects, and there was no observed heterogeneity within this group (*I* ^2^ = 28%). This suggests that combining BFR exercises may further enhance the PAP effect and optimize the CMJ vertical jump index, thus aligning with Cleary’s perspective.

#### 4.2.2 Exercise mode

An analysis of subgroups based on different exercise methods found that when compared to combining BFR training with low-intensity resistance exercise and high-intensity resistance training, combining BFR with body-weight exercises is more likely to induce PAP.

In traditional resistance training, effective improvements in muscle absolute strength typically requires an intensity of ≥70% 1RM (Ratame et al., 2009). However, the occurrence of the PAP phenomenon depends on post-training fatigue levels (Boullosa et al., 2013). Consequently, determining the optimal method to induce PAP is challenging, and for various intensity resistance exercises combined with BFR, participants may experience levels of fatigue greater than that induced by PAP. Additionally, Che et al. (2021) found that the subjective fatigue level during pressurized low-intensity resistance exercises, measured using RPE, was more pronounced in the pressurized training state.

The recruitment of high-threshold motor units is one of the mechanisms proposed by PAP, as observed by Chen et al. (2022); we found that during a 120-s warm-up at varying intensities, the higher the exercise intensity, the more pronounced the warm-up effect. Although more muscle fibers are recruited, contributing to enhanced muscle strength (Sweeney et al., 1993), BFR exercises combined with light loads (such as self-weight) may also trigger similar responses as heavy-load exercises. For instance, Miller et al. (2018) discovered that in a study combining BFR with whole-body vibration, both whole-body vibration and maximum isometric contraction exercises significantly improved vertical jump height under BFR conditions. This indicates that BFR training can yield comparable effects to high-load training, even with lower training intensity, thereby reducing the risk of sports injuries and excessive exercise loads.

#### 4.2.3 Compressive strength

With the change in pressure intensity, the induction degree of PAP is also different. When compared to no BFR training conducted conditions, lower limb occlusion above the brachial artery systolic pressure induced a significant amount of PAP stimulation. However, intramuscular hypoxia caused by high-intensity compression easily accelerated the fatigue response of muscle fibers (García-Pinillos et al., 2015).

A study also showed that low-intensity exercise with BFR pressure hardly induces the body’s stress response (Wang, 2021). High-intensity BFR pressure can lead to anaerobic metabolism, which results in lactic acid accumulation and a significant decrease in muscle torque. On the other hand, excessive AOP can easily cause severe limb ischemia and cardiovascular adverse events due to muscle edema (Spranger et al., 2015). Therefore, it is recommended to use BFR exercise with 40%∼60% AOP for immediate muscle strength growth.

### 4.3 Study limitations

The 18 articles included were not blinded and the methodology is limited in this respect. It is difficult to use blinding in this type of study because of the corresponding ethical requirement for human subjects to sign an informed consent form. In the few articles retrieved for this study, the authors could not be contacted to obtain the required data, which is theoretically biased, but the authors do not believe that this would have a subversive effect on the results of this study. Limitations in the research field and theme, such as the small number of studies evaluating lower extremity muscle function, the generally low sample sizes, and variations in intervals after BFR exercises among studies, may have introduced potential bias. Furthermore, despite extensive research, there remains a shortage of large-scale, high-quality studies on BFR training. Future studies should improve the credibility of the study by expanding the sample size as much as possible by improving the experimental design. For PAP induction, the effects of the training status, exercise interval time, and other factors on the PAP effect should be further clarified.

## 5 Conclusion

BFR exercises induce lower limb muscle activation and PAP effects to overcome self-weight. BFR exercises with 40%–60% AOP are more likely to induce PAP. BFR exercises in combination with a warm-up to overcome dead weight help improve the height of the CMJ vertical jump.

## Data Availability

The original contributions presented in the study are included in the article/Supplementary material; further inquiries can be directed to the corresponding author.
